# Supplementary material for: Biweekly CAPOX versus triweekly CAPOX in the adjuvant therapy of post-surgery CRC: A randomized controlled trial
Source: PLoS One. 2025 Jul 11;20(7):e0313472. doi: 10.1371/journal.pone.0313472 (PMC12250227; doi:10.1371/journal.pone.0313472)
Supplement: S2 File — (PDF) [file pone.0313472.s002.pdf]

肠癌术后 2 周及 3 周 XELOX 辅助化疗的疗效及安全性前瞻性研究方案

组长单位：浙江大学医学院附属第一医院

项目负责人：陈文斌

承担科室：结直肠外科

联系电话：13857196863

参加单位：浙江大学医学院附属第一医院

研究年限：2018 年 05 月-2022 年 12 月

版本号：V1.0

版本日期：2018 年 05 月 01 日

方 案 摘 要（限 1 页）

**项目名称**	肠癌术后2周及3周XELOX辅助化疗的疗效及安全性前瞻性研究
**关键词（3-5 个）**	肠癌；辅助化疗；modified XELOX
**研究目的**	探索2周XELOX辅助化疗的疗效及安全性
**研究设计**	随机对照，单中心，开放标签的前瞻性研究
**计划样本量**	160例（每组各80例）
**研究人群定义**	须满足以下所有条件：<br>1. 经组织学或细胞学确诊的肠腺癌<br>2. 确诊后行根治性手术切除，切术后分期为高危 II 期或 III 期患者<br>3. 美国东部肿瘤合作组体力状况评分（ECOG PS）小于等于 2 分<br>4. 既往未行任何形式的化疗
**干预方案**	肠癌辅助治疗随机入组 2 周 XELOX 组和 3 周 XELOX 组
**研究终点**	主要终点：血小板下降率<br>次要终点：3 年无疾病生存时间（DFS）
	安全性：血液学毒性、消化道毒性、外周神经毒性
**特殊伦理说明（若有）**	无
**研究期限（预计起始和终止时间）**	2018年5月至2022年12月
**主要生物样本采集及保存计划**	无

一、研究背景

结直肠癌是人类最常见的恶性肿瘤之一，高危 II 期及 III 期结直肠癌患者行术后辅助化疗能有效降低术后复发率，延长结直肠癌患者无病生存时间。国际上多项研究已经证实 xelox 方案是肠癌术后辅助治疗的标准治疗[1-5]。事实上，结直肠癌术后辅助因为化疗毒副反应较大，很大一部分患者无法坚持足疗程化疗，临床上观察到将标准 xelox 方案改良为 2 周 xelox 方案能明显下降化疗副反应，既往在胃癌中也有类似研究证实 2 周方案可以明显下降化疗毒副反应[6]，进一步提高患者完成术后辅助治疗疗程的完整性。

然而，目前针对 2 周 xelox 和 3 周 xelox 方案对比治疗肠癌术后患者的经验仍然非常有限，最佳的术后辅助治疗方案仍待于进一步探索。我们中心既往已有长期行 2 周及 3 周 xelox 方案治疗的经验，但缺乏前瞻性研究证实其疗效。因此，本项研究采取前瞻性设计，对比这两种辅助治疗方案的优劣性及安全性方案的差异，旨在探索出更佳的辅助治疗方案。

二、研究目的

1. 主要目的：2 周 XELOX 方案及 3 周 XELOX 方案的血液学毒性（血小板下降）
2. 次要目的：2 周 XELOX 方案及 3 周 XELOX 方案的 3 年 DFS（disease free survival）

三、研究设计类型、原则与试验步骤

这是一项前瞻性随机对照开放标签的研究，通过区组随机化分组，拟入组 160 例患者，每组分别 80 例。

实验步骤：

筛选期：通过筛选，符合入选标准且不符合排除标准的患者将进入本研究。并且在病例报告表（CRF）中评价和记录下列数据：患者基本资料（年龄、性别、种族、身高、体重、体表面积）；ECOG 评分；血常规检查结果；生化检查结果。

病理学结果

治疗期：筛选入组后随机入组 2 周及 3 周 XELOX 治疗组，并在每个化疗周期完成如下的评估和步骤，并记录在 CRF（病例报告表）中：血常规检查，每周 2 次检测。生化检查，每周 1 次检测。不良反应的发生率及严重程度。

随访期：每 3 个月进行一次影像学复查，排除复发转移；生存期随访。

四、研究人群选择

1. 入选标准

1. 经组织学或细胞学确诊的肠腺癌

2. 确诊后行根治性手术切除，切术后分期为高危 II 期或 III 期患者
3. 美国东部肿瘤合作组体力状况评分（ECOG PS）小于等于 2 分
4. 既往未行任何形式的化疗
5. 中性粒细胞≥1.5×109/L，血小板>100×109/L，血红蛋白≥9g/dL;
6. 肝功能转氨酶≤2.5ULN(如合并肝转移≤5ULN)，碱性磷酸酶≤2.5ULN(如合并肝转移≤5ULN，合 并骨转移≤10ULN)，总胆红素<1.5ULN

2. 排除标准

1. 目前有难以控制的感染，或接受化疗前的 72 h 内接受过系统抗生素治疗；
2. 任何骨髓异常增生及其他造血功能异常者；
3. 5 年内患有其他恶性肿瘤（原位癌、基底细胞癌等除外）；
4. 发生中枢神经系统转移患者；
5. 对本研究中的治疗药物过敏者；
6. 患有精神或神经系统疾患，不能配合者；
7. 妊娠或哺乳期女性患者；育龄期妇女拒绝接受避孕措施者；
8. 有活动性的自身免疫性疾病、自身免疫性疾病史，正在使用皮质类固醇激素类药物或免疫抑制剂，或者正在使用激素替代治疗，如甲状腺素、胰岛素等。
9. 首次给药前 30 天内接种过活疫苗（允许使用注射型季节性流感疫苗，因该疫苗为灭活疫苗）
10. 患其他疾病不适合入组者，如活动性肺结核、乙肝（经治疗后乙肝病毒滴度 HBV-DNA<500IU/ml，且肝功能正常则可入组）、丙肝病毒检测阳性、无法纠正的电解质紊乱、不可控制的心包积液、胸腔积液及腹腔积液等；
11. 筛选期 30 天内曾接受其他任何试验药物治疗或参加过另一项干预性临床试验；
12. 有器官移植病史；
13. 研究者认为不适合入组者。

3．剔除标准

1) 试验期间同时接受任何其它研究性或其他任何抗癌治疗，包括化疗、放疗、生物疗法，也包括使用中成药来进行抗癌治疗；

2) 依从性差者；

3) 不符合入选/排除标准；

4) 试验前后主要检测指标不全者；

5) 未用药。

4. 终止研究标准

1) RECIST1.1 定义的疾病进展或临床进展；
2) 对症治疗，延迟给药或剂量调整后，仍无法控制的不可耐受的不良事件；
3) 经过最佳支持治疗后，按方案调整剂量 2 次后仍然复发的 3/4 级毒性；
4) 治疗延迟超过 21 天；
5) 存在影响继续接受研究治疗的并发症；
6) 给药开始后，发现不符合入选标准或与排除标准相抵触等，必须从试验对象中除外的；
7) 严重违反临床试验方案的；
8) 不能遵循方案进行治疗、依从性差者；
9) 遵守临床试验方案有困难的；
10) 受试者本人要求退出试验的；

五、研究方法与技术路线

1. 研究用药名称和规格

奥沙利铂（汇铂） 50mg / 支；卡培他滨（卓仑） 500mg / 片

2. 干预方案

实验组：2 周 XELOX ：奥沙利铂 85mg / m2 d1；卡培他滨 1000mg / m2 d1-10；q2w
对照组：3 周 XELOX ：奥沙利铂 130mg / m2 d1；卡培他滨 1000mg / m2 d1-14；q3w

六、观察指标

疗效评价：主要疗效指标：DFS：手术后随访至疾病复发进展的时间。次要疗效指标：OS：手术后随访至患者因任何原因死亡的时间。

安全性评价：包括骨髓抑制血液毒性、神经毒性以及消化道毒性

七、疗效评定标准

疾病进展评定按照 RECIST 1.1 对肿瘤影像学进行评估,每 3 个月进行 1 次，并记录。安全性评价按通用毒性标准（NCI-CTCAE 5.0）进行。将受试者生成所有死亡、所有 SAE 及药物有关的 AE 列表。神经毒量表评估神经毒性，用药阶段每周期评估 1 次。

八、不良事件的观察

不良事件（Adverse Event）：是指病人或临床试验受试者接受一种药品后出现的不良医学事件，但并不一定与治疗有因果关系。

严重不良事件（Serious Adverse Event）：临床试验过程中因使用药品引起以下损害情形之一的反应：（1）导致死亡；（2）危及生命；（3）致癌、致畸、致出生缺陷；（4）导致显著的或者永久的人体伤残或者器官功能的损伤；（5）导致住院或者住院时间延长；（6）导致其他重要医学事件，如不进行治疗可能出现上述所列情况的。

药物不良反应：是指在按规定剂量正常应用药品的过程中产生的有害而非所期望的、但又与药品应用有因果关系的反应。在一种新药或药品新用途的临床试验中，其治疗剂量尚未确定时，所有有害而非所期望的、与药品应用有因果关系的反应，均应视为药品不良反应

不良事件的观察及处理

观察与记录：研究者应认真观察受试者在临床研究期间发生的任何不良事件，要求受试者如实反映用药后的病情变化，避免诱导性提问。在观察疗效的同时注意观察不良反应或未预料到的毒副作用（包括症状、体征及实验室检查）。无论不良事件是否与试验药物有关均应在 CRF 表中详细记录，包括不良反应出现时间、症状、体征、程度、持续时间、实验室检查指标、处理方法、经过、结果、随访时间等，并应详细记录合并用药的情况，以便分析不良事件与试验药物相关性，记录时应签名并注明日期。

不良事件与研究药物相关性评估

研究者应按照受试者发生的不良事件的具体情况和受试者的既往病史、伴发病情况以及伴随用药等情况进行综合分析，判断不良事件与药物的关系。研究者应对受试者在用药过程中出现的症状进行因果分析，以便对不良事件和试验药物之间可能存在的关联作出评估

九、研究的质量控制与质量保证

本研究将建立一个独立的数据监查小组，其目的是对整个研究过程中的安全性事件和疗效进行审核。为了审核安全性事件和疗效结果，小组成员需要查看诊断和处理研究经过相关的医疗原始记录。数据监查小组还负责审核研究者提交给申办者的其他文件，比如严重不良事件报告 SAE 以及随 SAE 报告递交的医疗原始记录，或临床结果病例报告表 (CRF)，以及时发现是否存在安全性风险。

十、研究结果分析计划

统计分析计划将在研究结束前开始制定，并于数据库锁定前确定。计划将提供所有统计

学分析将要涉及的内容，包括用于分析的数据集的定义和数据的衍生算法，针对不同指标采用的统计学描述和分析方法。

对各基线指标（如病史等）及人口学特征指标进行总结，计量指标用例数、均值、标准差、中位数、最小值和最大值进行描述。计数指标用例数、百分数进行描述。对总的入选例数、各研究人群入选例数进行描述。并对各种未完成情况进行总结。

全分析集（Full Analysis Set，FAS）：包括所有随机化入组的受试者。而且至少服用过一次药物，并有至少一次随访记录者。

接受治疗人群（ATP）：该人群包括接受治疗的所有患者。该类人群是评估给药治疗、依从性和安全性的主要人群。

可评估人群：该人群包括 ATP 患者中进行了基线评估和至少有两个治疗周期评估的人群，足以对相应的疗效终点进行评估。

十一、临床研究的伦理学

临床研究将遵循世界医学大会《赫尔辛基宣言》等相关规定。在研究开始之前，由伦理委员会批准该试验方案后才实施临床研究。每一位受试者入选本研究前，研究者有责任向受试者或其代理人完整、全面地介绍本研究的目的、程序和可能的风险，并签署书面知情同意书，应让受试者知道他们有权随时退出本研究，知情同意中应作为临床研究文件保留备查。研究过程中将保护受试者的个人隐私与数据机密性。

十二、研究时间安排

2018-5 至 2021-12 入组 160 例患者

2021-12 至 2024-10 数据处理和资料总结，主要对临床试验所得到的资料进行录入、核查、统计、总结，完成临床研究总结报告。生存期随访，数据补充和完善。

十三、参加人员

姓名	职称/专业	分工
陈文斌	主任医师 / 结直肠外科	项目负责人
方维佳	主任医师 / 肿瘤内科	项目策划
王丹阳	副主任医师 / 结直肠外科	病例收集

张航瑜	主治医师 / 肿瘤内科	病例随访
杨天兴	主任医师 / 肿瘤内科	病例收集及随访
余佳泽	主治医师 / 肿瘤内科	病例收集及随访

十四、主要参考文献

1. Compton CC, Fielding LP, Burgart LJ, Conley B, Cooper HS, Hamilton SR, Hammond ME, Henson DE, Hutter RV, Nagle RB *et al*: **Prognostic factors in colorectal cancer. College of American Pathologists Consensus Statement 1999**. *Arch Pathol Lab Med* 2000, **124**(7):979-994.
2. Andre T, Boni C, Mounedji-Boudiaf L, Navarro M, Tabernero J, Hickish T, Topham C, Zaninelli M, Clingan P, Bridgewater J *et al*: **Oxaliplatin, fluorouracil, and leucovorin as adjuvant treatment for colon cancer**. *N Engl J Med* 2004, **350**(23):2343-2351.
3. Andre T, Boni C, Navarro M, Tabernero J, Hickish T, Topham C, Bonetti A, Clingan P, Bridgewater J, Rivera F *et al*: **Improved overall survival with oxaliplatin, fluorouracil, and leucovorin as adjuvant treatment in stage II or III colon cancer in the MOSAIC trial**. *J Clin Oncol* 2009, **27**(19):3109-3116.
4. Andre T, de Gramont A, Vernerey D, Chibaudel B, Bonnetain F, Tijeras-Raballand A, Scriva A, Hickish T, Tabernero J, Van Laethem JL *et al*: **Adjuvant Fluorouracil, Leucovorin, and Oxaliplatin in Stage II to III Colon Cancer: Updated 10-Year Survival and Outcomes According to BRAF Mutation and Mismatch Repair Status of the MOSAIC Study**. *J Clin Oncol* 2015, **33**(35):4176-4187.
5. Schmoll HJ, Tabernero J, Maroun J, de Braud F, Price T, Van Cutsem E, Hill M, Hoersch S, Rittweger K, Haller DG: **Capecitabine Plus Oxaliplatin Compared With Fluorouracil/Folinic Acid As Adjuvant Therapy for Stage III Colon Cancer: Final Results of the NO16968 Randomized Controlled Phase III Trial**. *J Clin Oncol* 2015, **33**(32):3733-3740.
6. Kuo YC, Liu HT, Lin YL, Yang YC, Yang TS, Liau CT, Shen WC, Hsu HC, Chou WC, Chen JS: **Modified biweekly oxaliplatin and capecitabine for advanced gastric cancer: a retrospective analysis from a medical center**. *Biomed J* 2014,

37(3):141-146.
