# Supplementary material for: Biweekly CAPOX versus triweekly CAPOX in the adjuvant therapy of post-surgery CRC: A randomized controlled trial
Source: PLoS One. 2025 Jul 11;20(7):e0313472. doi: 10.1371/journal.pone.0313472 (PMC12250227; doi:10.1371/journal.pone.0313472)
Supplement: S3 File — (PDF) [file pone.0313472.s003.pdf]

Prospective study of efficacy and safety of biweekly XELOX and triweekly XELOX adjuvant chemotherapy after colorectal cancer surgery

Group leader: The First Affiliated Hospital of Zhejiang University School of Medicine

Project leader: Wenbin Chen

Department: Colorectal Surgery

Contact number: 13857196863

Participant: The First Affiliated Hospital of Zhejiang University School of Medicine

Study period: May 2018 - December 2022

Version number: V1.0

Release date: May 1, 2021

Abstract

Key words (3-5): Colorectal cancer; Adjuvant chemotherapy; modified XELOX

**Project name**	Prospective study of efficacy and safety of biweekly XELOX and triweekly XELOX adjuvant chemotherapy after colorectal cancer surgery

**Objectives of the study**	To explore the efficacy and safety of 2-week XELOX adjuvant chemotherapy
**Study design**	This was a randomized, controlled, open-label prospective study
**Sample size**	160 cases (80 cases in each group)
**Patient population**	All of the following conditions must be met:<br>1. Adenocarcinoma of the bowel confirmed histologically or cytologically<br>2. After diagnosis, radical surgical resection was performed, and patients were classified as high-risk stage II or III after resection<br>3. The Eastern Cooperative Oncology Group performance status (ECOG PS) score was less than or equal to 2<br>4. He had not previously received any form of chemotherapy
**Intervention regimen**	Adjuvant therapy for colorectal cancer was randomized to biweekly or triweekly XELOX
**Study end points**	Primary endpoint: thrombocytopenia<br>Secondary end point: 3-year DFS
**Special ethical statement (if any)**	N/A

**Study duration (estimated start and end times)**	May 2018 to December 2022
**Major biosample collection and preservation programs**	N/A

I. Research background

Colorectal cancer is one of the most common human malignancy, phase II and III colorectal cancer patients at high risk of postoperative adjuvant chemotherapy can effectively reduce the postoperative recurrence rate, prolong disease-free survival time of patients with colorectal cancer. A number of international studies have confirmed that xelox program is the standard treatment for postoperative adjuvant therapy of colorectal cancer [1-5]. In fact, due to the high toxic and side effects of postoperative adjuvant chemotherapy for colorectal cancer, a large proportion of patients cannot adhere to the full course of chemotherapy. It has been observed in clinical practice that the standard xelox regimen can significantly reduce the side effects of chemotherapy. Similar studies in gastric cancer have confirmed that the 2-week regimen can significantly reduce the toxic and side effects of chemotherapy [6]. To further improve the postoperative adjuvant therapy in patients with complete course of integrity.

However, at present the xelox for 2 weeks and 3 weeks xelox scheme comparison of the very limited experience in the therapy of patients with colorectal surgery postoperative adjuvant therapy of best scheme is still to be in further exploration. Our center always has long line 2 weeks and 3 weeks of xelox regimen for experience, but the lack of prospective studies to confirm their effectiveness. Therefore, a prospective study was conducted to compare the advantages and disadvantages of these two adjuvant regimens and the differences in safety regimens, aiming to explore a better adjuvant regimen.

II. Objectives of the study

1. Primary objective: Hematologic toxicity (thrombocytopenia) of 2-week and 3-week XELOX regimens

2. Secondary objective: 3-year disease-free survival (DFS) of 2-week and 3-week XELOX regimens.

III. Study design, principles, and procedures

This was a prospective randomized controlled open-label study. A total of 160 patients were enrolled by block randomization, with 80 patients in each group.

Experimental procedures:

Screening stage: through screening, in line with the inclusion criteria and do not accord with standard of exclusion of patients will go into this study. And in the case report form (CRF) in the evaluation and record the following data: basic data (age, gender, race, height, body weight, body surface area); ECOG score; Blood routine test results; Results of biochemical tests. Pathological results

Treatment period: patients were randomly assigned to the 2-week and 3-week XELOX treatment groups after screening and enrollment and completed the following assessment and steps in each chemotherapy cycle and recorded in the CRF (case report form): blood routine examination, testing twice a week. Biochemical tests were performed once a week. The incidence and severity of adverse reactions were recorded.

During the follow-up period, imaging reexamination was performed every 3 months to exclude recurrence and metastasis. Survival time was followed up.

IV. Study population selection

1. Inclusion criteria

1. Histologically or cytologically confirmed adenocarcinoma of the bowel

2. After diagnosis, radical surgical resection was performed, and patients were classified as high-risk stage II or III after resection

3. The Eastern Cooperative Oncology Group performance status (ECOG PS) score was less than or equal to 2

4. Not previously received any form of chemotherapy

5. Neutrophil ≥1.5×109/L, platelet >100×109/L, hemoglobin ≥9g/dL.

6. Liver function transaminase ≤2.5ULN (if combined with liver metastasis ≤5ULN), alkaline phosphatase ≤2.5ULN (if combined with liver metastasis ≤5ULN, combined with bone metastasis ≤10ULN), total bilirubin <1.5ULN

2. Exclusion criteria

1. Current infection that is difficult to control, or systemic antibiotic treatment within 72 hours before chemotherapy.

2. Any bone marrow dysplasia or other hematopoietic dysfunction.

3. Other malignant tumors within 5 years (except carcinoma in situ, basal cell carcinoma, etc.).

4. Patients with central nervous system metastasis.

5. Patients who were allergic to the treatment drugs in this study.

6. Patients with a mental or neurological disorder who are unable to cooperate.

7. Pregnant or lactating women; Women of childbearing age who refuse to accept contraception.

8. Active autoimmune disease, history of autoimmune disease, current use of corticosteroids or immunosuppressants, or current use of hormone replacement therapy such as thyroxine, insulin, etc.

9. Have received live vaccine within 30 days before the first dose (injectable seasonal influenza vaccine is allowed as it is inactivated)

10. Patients with other diseases were not eligible for enrollment, such as active tuberculosis, hepatitis B (after treatment, hepatitis B virus titer HBV-DNA<500IU/ml, and normal liver function), positive hepatitis C virus test, uncorrected electrolyte disturbance, uncontrollable pericardial effusion, pleural effusion, and peritoneal effusion.

11. Had received any other trial drug or had participated in another interventional clinical trial within 30 days of the screening period.

12. A history of organ transplantation.

13. And those deemed by the investigator to be ineligible for enrollment.

3. Exclusion Criteria

1) receiving any other investigational or any other anticancer treatment simultaneously during the trial period, including chemotherapy, radiotherapy, biological therapy, and the use of Chinese patent medicine for anticancer treatment.
2) patients with poor compliance.
3) did not meet the inclusion/exclusion criteria;
4) patients with incomplete main laboratory indicators before and after the trial; 5) no medication.

4. Study termination criteria

1) disease progression or clinical progression according to RECIST1.1;
2) symptomatic treatment, or intolerable adverse events that could not be controlled after delayed administration or dose adjustment;
3) Grade 3/4 toxicities that recurred after two dose adjustments according to the protocol after best supportive care;
4) treatment delay more than 21 days;
5) the presence of complications that would prevent continuation of the study treatment;
6) After the initiation of drug administration, the subjects must be excluded from the study if they do not meet the inclusion criteria or exclusion criteria;
7) serious violation of the clinical trial protocol;
8) unable to follow the protocol for treatment, poor compliance;
9) having difficulty complying with the clinical trial protocol;
10) withdrawal from the trial at the request of the subject;

V. Research methods and technical route

1. Name and specifications of investigational drugs

Oxaliplatin 50mg/dose; Capecitabine (Zolam) 500mg/tablet

2. Intervention protocol

Experimental group: 2 weeks XELOX: oxaliplatin 85mg/m2 d1; Capecitabine 1000mg/m2 d1-10; q2w
Control group: 3 weeks XELOX: oxaliplatin 130mg/m2 d1; Capecitabine 1000mg/m2 d1-14; q3w

VI. Observation indicators

Efficacy evaluation: DFS: time from surgery follow-up to disease recurrence and progression.

Safety evaluation included myelosuppression, hematologic toxicity, neurotoxicity, and gastrointestinal toxicity.

VII. Criteria for efficacy evaluation

Tumor progression was evaluated according to RECIST 1.1, once every 3 months and recorded. Safety was evaluated according to the Common toxicity Criteria (NCI-CTCAE 5.0). A list of all deaths, all SAEs, and drug-related AEs was generated for the subjects. The neurotoxicity scale was used to assess neurotoxicity, which was assessed once per cycle during the dosing phase.

VIII. Observation of adverse events

Adverse events (Adverse Event): refers to the patient or clinical trial subjects occurred after a drug Adverse medical events but does not necessarily have causal relationship with treatment.

Serious Adverse Event: a reaction that results from the use of a drug in the course of a clinical trial that causes one of the following adverse events: (1) death; (2) life-threatening; (3) carcinogenic, teratogenic and birth defects; (4) causing significant or permanent disability or impairment of organ function; (5) resulting in hospitalization or prolonged hospitalization; (6), leading to other important medical event, such as no treatment may be listed in the above case.
Adverse drug reaction (ADR) refers to a harmful and undesired reaction that occurs during the normal application of a drug at the prescribed dose, but is causally related to the application of the drug. In the clinical trials of a new drug or drug use, the treatment dose has

not yet been determined, all harmful rather than expected, and drug application causation of reaction, shall be considered as adverse drug reactions

Observation and management of adverse events

Observation and recording: investigators should carefully observe any adverse events that occur in the subjects during the clinical study, ask the subjects to truely reflect the changes in the condition after medication, and avoid leading questions. In the observation of curative effect at the same time observe adverse reactions or unexpected side effects, including symptoms, signs and laboratory examination). Whether adverse events associated with experimental drug shall be in CRF detailed records in the table, including adverse reaction time, symptoms, signs, degree, duration, laboratory examination indexes, processing method, after, results and follow-up time, etc., and should be a detailed record of drug combination, so that the correlation analysis of adverse events and experimental drugs, The records should be signed and dated.

Adverse events were assessed for association with study medication

Researchers should comprehensively analyze the adverse events and the past medical history, concomitant diseases and concomitant medications of the subjects to judge the relationship between adverse events and drugs. Investigators should conduct a causal analysis of the subjects' symptoms during the course of medication in order to evaluate the possible association between adverse events and trial drugs

IX. Quality control and assurance of the study

This research will establish an independent data monitoring group, its purpose is to research on the security event and curative effect in the process of review. To review safety events and efficacy results, panel members were required to have access to the relevant medical source records of the diagnostic and management trials. The data-monitoring team also reviewed other documents submitted by the investigators to the sponsor, such as the SAEs for serious adverse events and the medical records submitted with the SAEs or the case report forms (CRFS) for clinical results, to identify any safety risks in a timely manner.

X. Results Analysis Plan

Development of the statistical analysis plan will begin before the end of the study and will be determined before the database lock. The plan will provide all aspects of the statistical analysis, including the definition of the data set used for the analysis and the algorithms for deriving the data, the statistical description and analysis methods for the various measures.

The baseline indicators (such as medical history, etc.) and demographic characteristics indicators were summarized, and the number of cases, mean, standard deviation, median, minimum and maximum values of measurement indicators were described. The enumeration indicators were described by the number of cases and percentage. The total number of enrolled cases and the number of enrolled cases in each study population were described. And to summarize various outstanding cases.

Full Analysis Set (FAS): All subjects who were randomized to the study group were included. And at least once drugs and have at least one follow-up records.
Population on Treatment (ATP): This population included all patients who received

treatment. This population is the primary population for the assessment of medication administration, adherence, and safety.

Evaluable population: This population included patients with ATP who had a baseline assessment and at least two treatment cycles of assessment and was sufficient for the assessment of the respective efficacy end points.

XI. Ethics of clinical Research

Clinical research shall follow the Declaration of Helsinki of the World Medical Assembly and other relevant regulations. The clinical study will be carried out after the approval of the trial protocol by the ethics committee prior to the initiation of the study. Before every subjects were included in this study, the researchers have responsibility to the participants or its agent complete, comprehensive introduction to the purpose of this study, application and potential risks, and sign a written informed consent, should let the participants know that they have the right to exit this study at any time, informed consent should be as clinical research documents retained for future reference. The personal privacy and data confidentiality of the subjects will be protected during the study.

XII. research schedule

A total of 160 patients were enrolled from May 2018 to December 2021
Data processing and data summary from 2021-December to 2024-October, mainly input, check, statistics and summary of the data obtained from clinical trials and complete the clinical study summary report. Survival follow-up, data supplement and improvement.

XIII. PI Name

Chen Wenbin and Weijia Fang.

Job title/major

Chief physician/Colorectal Surgery Chief Physician/Medical Oncology

Division of labor

Project Leader Project planning

Sub-I Name

Wang Danyang

Hang Yu Zhang

Yang Tianxing

Yu Jia-ze

Associate chief physician/Colorectal Surgery Attending physician/Medical Oncology

Chief Physician/Medical Oncology Attending physician/Medical Oncology

References

1. Compton CC, Fielding LP, Burgart LJ, Conley B, Cooper HS, Hamilton SR, Hammond ME, Henson DE, Hutter RV, Nagle RB et al: Prognostic factors in colorectal cancer.

College of American Pathologists Consensus Statement 1999. Arch Pathol Lab Med 2000, 124(7):979-994.

2. Andre T, Boni C, Mounedji-Boudiaf L, Navarro M, Tabernero J, Hickish T, Topham C, Zaninelli M, Clingan P, Bridgewater J et al: Oxaliplatin, fluorouracil, and leucovorin as adjuvant treatment for colon cancer. N Engl J Med 2004, 350(23):2343-2351.
3. Andre T, Boni C, Navarro M, Tabernero J, Hickish T, Topham C, Bonetti A, Clingan P, Bridgewater J, Rivera F et al: Improved overall survival with oxaliplatin, fluorouracil, and leucovorin as adjuvant treatment in stage II or III colon cancer in the MOSAIC trial. J Clin Oncol 2009, 27 (19) : 3109-3116.
4. Andre T, de Gramont A, Vernerey D, Chibaudel B, Bonnetain F, Tijeras-Raballand A, Scriva A, Hickish T, Tabernero J, Van Laethem JL et al: Adjuvant Fluorouracil, Leucovorin, and Oxaliplatin in Stage II to III Colon Cancer: Updated 10-Year Survival and Outcomes According to BRAF Mutation and Mismatch Repair Status of the MOSAIC Study. J Clin Oncol 2015, 33(35):4176-4187.
5. Schmoll HJ, Tabernero J, Maroun J, de Braud F, Price T, Van Cutsem E, Hill M, Hoersch S, Rittweger K, Haller DG: Capecitabine Plus Oxaliplatin Compared With Fluorouracil/Folinic Acid As Adjuvant Therapy for Stage III Colon Cancer: Final Results of the NO16968 Randomized Controlled Phase III Trial. J Clin Oncol 2015, 33(32):3733-3740.
6. Kuo YC, Liu HT, Lin YL, Yang YC, Yang TS, Liau CT, Shen WC, Hsu HC, Chou WC, Chen JS: Modified biweekly oxaliplatin and capecitabine for advanced gastric cancer: a retrospective analysis from a medical center. Biomed J 2014, 37(3):141-146.
