# Supplementary material for: Biweekly CAPOX versus triweekly CAPOX in the adjuvant therapy of post-surgery CRC: A randomized controlled trial
Source: PLoS One. 2025 Jul 11;20(7):e0313472. doi: 10.1371/journal.pone.0313472 (PMC12250227; doi:10.1371/journal.pone.0313472)
Supplement: Attachment — Submitted filename: comments 14.docx [file pone.0313472.s004.docx]

The study titled "Biweekly CAPOX versus Triweekly CAPOX in the adjuvant therapy of post-surgery CRC: a randomized controlled trial" is a well-designed and clinically relevant investigation that addresses an important question in the management of colorectal cancer (CRC). The authors have conducted a randomized controlled trial to compare the safety and efficacy of a modified biweekly CAPOX regimen with the conventional triweekly CAPOX regimen in high-risk stage II and stage III post-surgery CRC patients. The study is particularly commendable for its focus on reducing treatment-related adverse events, such as thrombocytopenia and neutropenia, while maintaining therapeutic efficacy. The findings suggest that the biweekly CAPOX regimen is associated with fewer hematological adverse events and comparable 3-year disease-free survival (DFS) rates, which could have significant implications for improving patient tolerance and adherence to adjuvant chemotherapy. The study is well-written, methodologically sound, and provides valuable insights into optimizing adjuvant chemotherapy for CRC patients.

**Comments for Revision:**

1. While the study mentions that the sample size was calculated based on the primary outcome of thrombocytopenia, it would be beneficial to provide more details on the assumptions used for this calculation (e.g., expected incidence rates, effect size, power, and significance level). This will help readers understand the robustness of the study design and whether the sample size was adequate to detect clinically meaningful differences.
2. The study reports a median follow-up period of 42 months, which is commendable. However, it would be useful to discuss whether this follow-up duration is sufficient to capture long-term outcomes, particularly for DFS and overall survival (OS). Consider providing a rationale for the chosen follow-up period and whether longer follow-up is planned.
3. The study briefly mentions subgroup analyses based on treatment duration (3-month vs. 6-month). It would be valuable to provide more detailed results of these subgroup analyses, particularly in terms of adverse events and DFS. This could help identify specific patient populations that may benefit more from the biweekly regimen.
4. The study uses Kaplan-Meier estimation for DFS analysis and Cox proportional hazard models for hazard ratios (HRs). It would be helpful to clarify whether any adjustments were made for multiple comparisons, especially given the secondary endpoints. Additionally, consider discussing the potential impact of confounding variables on the results and whether any multivariate analyses were performed.
5. While the study reports a lower incidence of thrombocytopenia and neutropenia in the biweekly group, it would be beneficial to provide more detailed information on the severity and management of these adverse events. For example, how many patients required dose reductions, treatment delays, or supportive care interventions?
6. The discussion section could be expanded to include a more detailed discussion of the clinical implications of the findings. For instance, how might the reduced incidence of hematological adverse events impact patient quality of life, treatment adherence, and overall healthcare costs? Additionally, consider discussing how these findings might influence clinical guidelines or practice.
7. The study acknowledges that it is a pilot study with a limited number of patients. It would be helpful to elaborate on other potential limitations, such as the single-center design, potential selection bias, and the generalizability of the findings to other populations or settings.
8. The conclusion could be strengthened by suggesting directions for future research. For example, are there plans for a larger, multicenter trial to confirm these findings? Could future studies explore the use of biomarkers or molecular profiling to further personalize adjuvant chemotherapy regimens?
