# Supplementary material for: Biweekly CAPOX versus triweekly CAPOX in the adjuvant therapy of post-surgery CRC: A randomized controlled trial
Source: PLoS One. 2025 Jul 11;20(7):e0313472. doi: 10.1371/journal.pone.0313472 (PMC12250227; doi:10.1371/journal.pone.0313472)
Supplement: Attachment — Submitted filename: Response to Reviewers.docx [file pone.0313472.s006.docx]

# Response to Journal Requirements

Manuscript ID: PONE-D-24-47321

Title: Biweekly CAPOX versus Triweekly CAPOX in the adjuvant therapy of post-surgery CRC: a randomized controlled trial

We sincerely thank the editor for the time and effort dedicated to reviewing our manuscript. We truly appreciate your thoughtful comments and the opportunity to revise our work in accordance with the journal's requirements. We are also very pleased about the possibility of publishing our work in *PLOS ONE*.

1. Please ensure that your manuscript meets PLOS ONE's style requirements, including those for file naming. The PLOS ONE style templates can be found at https://journals.plos.org/plosone/s/file?id=wjVg/PLOSOne_formatting_sample_main_body.pdf and <https://journals.plos.org/plosone/s/file?id=ba62/PLOSOne_formatting_sample_title_authors_affiliations.pdf>.

**Response:**

Thank you for your comment. We have carefully reviewed and ensured that our manuscript meets all of PLOS ONE's style requirements, including those for file naming. We have referred to the provided templates and make several necessary adjustments to align with the journal's formatting guidelines.

2. Thank you for submitting your clinical trial to PLOS ONE and for providing the name of the registry and the registration number. The information in the registry entry suggests that your trial was registered after patient recruitment began. PLOS ONE strongly encourages authors to register all trials before recruiting the first participant in a study.

As per the journal’s editorial policy, please include in the Methods section of your paper:

a) your reasons for your delay in registering this study (after enrolment of participants started);

b) confirmation that all related trials are registered by stating: “The authors confirm that all ongoing and related trials for this drug/intervention are registered.

**Response:**

Thank you for your helpful feedback. We acknowledge that the trial was registered after participant recruitment began. This delay was due to unforeseen logistical challenges, which led to the registration occurring after enrollment had already started. We have now included the following statement in the Methods section of our manuscript:

“The authors acknowledge that the trial was registered after participant recruitment started due to logistical delays. We confirm that all ongoing and related trials for this intervention are registered.”

This revision has been made in accordance with your editorial policy, and we appreciate your attention to this matter.

3. We note that your Data Availability Statement is currently as follows: [All relevant data are within the manuscript and its Supporting Information files.]

Please confirm at this time whether or not your submission contains all raw data required to replicate the results of your study. Authors must share the “minimal data set” for their submission. PLOS defines the minimal data set to consist of the data required to replicate all study findings reported in the article, as well as related metadata and methods (https://journals.plos.org/plosone/s/data-availability#loc-minimal-data-set-definition).

For example, authors should submit the following data:

- The values behind the means, standard deviations and other measures reported;

- The values used to build graphs;

- The points extracted from images for analysis.

Authors do not need to submit their entire data set if only a portion of the data was used in the reported study.

If your submission does not contain these data, please either upload them as Supporting Information files or deposit them to a stable, public repository and provide us with the relevant URLs, DOIs, or accession numbers. For a list of recommended repositories, please see https://journals.plos.org/plosone/s/recommended-repositories.

If there are ethical or legal restrictions on sharing a de-identified data set, please explain them in detail (e.g., data contain potentially sensitive information, data are owned by a third-party organization, etc.) and who has imposed them (e.g., an ethics committee). Please also provide contact information for a data access committee, ethics committee, or other institutional body to which data requests may be sent. If data are owned by a third party, please indicate how others may request data access.

**Response:**

Thank you for your comment regarding the Data Availability Statement. We confirm that the submission contains all the raw data required to replicate the results of the study. As per PLOS ONE’s data availability policy, the minimal data set includes the values behind the means, standard deviations, and other reported measures, as well as the data used to build graphs and points extracted from images for analysis.

We have uploaded the relevant data as Supporting Information files. We have also included a detailed explanation in the Data Availability section regarding the minimal data set required to replicate the study’s findings.

4. PLOS requires an ORCID iD for the corresponding author in Editorial Manager on papers submitted after December 6th, 2016. Please ensure that you have an ORCID iD and that it is validated in Editorial Manager. To do this, go to ‘Update my Information’ (in the upper left-hand corner of the main menu), and click on the Fetch/Validate link next to the ORCID field. This will take you to the ORCID site and allow you to create a new iD or authenticate a pre-existing iD in Editorial Manager.

**Response:**

Thank you for pointing this out. The first author and corresponding author now have ORCID iD and they have been validated in Editorial Manager. We have completed the process and can confirm that the ORCID iD is now included as required.

5. Thank you for stating the following in the Acknowledgments Section of your manuscript:

“This work was supported by Natural Science Foundation of Zhejiang Province, No. LQ23H160041.”

We note that you have provided additional information within the Acknowledgements Section that is not currently declared in your Funding Statement. Please note that funding information should not appear in the Acknowledgments section or other areas of your manuscript. We will only publish funding information present in the Funding Statement section of the online submission form.

Please remove any funding-related text from the manuscript and let us know how you would like to update your Funding Statement. Currently, your Funding Statement reads as follows:

“Zhejiang Provincial National Science Foundation of China LQ23H160041 (to H.Z)”

Please include your amended statements within your cover letter; we will change the online submission form on your behalf.

**Response:**

We appreciate your comment about the placement of funding information in the Acknowledgments section. As per your guidance, we have removed all funding-related text from the Acknowledgments section. The correct funding information has been added to the Funding Statement section of the manuscript, as follows:

“This work was supported by the Zhejiang Provincial National Science Foundation of China, No. LQ23H160041 (to H.Z.).”

This updated statement has been reflected in the Cover Letter, as requested.

6. Your ethics statement should only appear in the Methods section of your manuscript. If your ethics statement is written in any section besides the Methods, please delete it from any other section.

**Response:**

Thank you for your comment. We have revised the manuscript to ensure that the Ethics Statement only appears in the Methods section, as per the journal's requirements. Any instances of the ethics statement appearing outside of the Methods section have been removed.

# Response to Reviewers

Manuscript ID: PONE-D-24-47321

Title: Biweekly CAPOX versus Triweekly CAPOX in the adjuvant therapy of post-surgery CRC: a randomized controlled trial

We thank all the Reviewers for their valuable comments and suggestions. We have carefully revised the manuscript accordingly. Below are our point-by-point responses.

## Reviewer #1

Thank you for an interesting article that clearly outlines what was done and how to interpret the findings. I have a few comments to further clarify the article:

1. Comment 1:

Abstract: "This study aims" – please add an "s".

**Response:**

Thank you. We have corrected the phrase to "This study aims..." in the Abstract (line 25 of revised manuscript).

2. Comment 2:

Line 139: 0.44 effect size - isn't this a calculation based on a difference in proportions between the two groups? What proportions were used? was the proportion of all grade or grade 3+ the quantity of interest? Please provide a calculation that matches the incidence endpoint. Be clear if it is a rate estimated at any time during treatment, or if it is calculated from a time to event variable, which is possible if the AEs can take time to be experienced and there is differing times of follow up.

**Response:**

Thank you for pointing this out. We have clarified that the effect size was based on the difference in the incidence of all grade thrombocytopenia.

The sample size calculation was based on the primary outcome of all-grade thrombocytopenia. Drawing upon both previous literature^1^ and our center's clinical experience, we assumed an expected incidence of 60% for the triweekly CAPOX group. For the biweekly CAPOX group, based on preliminary pilot data and clinical observations, we estimated a reduction to 39%. The effect size (Cohen’s h = 0.44) was calculated using the arcsine transformation formula for two proportions. With a two-sided significance level of 0.05 and a power of 80%, we determined that 80 participants per group were required.

We confirm that adverse events were assessed from the start of treatment through 28 days after the last dose of CAPOX, consistent with standard AE monitoring windows in clinical trials. The primary outcome—incidence of all-grade thrombocytopenia—was calculated as a binary endpoint (event/no event) during this predefined observation period, not as a time-to-event variable. This definition has now been clarified in the Methods section (lines 130-135 of revised manuscript).

"Adverse events were monitored from the first day of treatment initiation until 28 days after the last dose of CAPOX. All events were assessed according to the Common Terminology Criteria for Adverse Events Version (CTCAE) version 4.0, and the incidence of all-grade thrombocytopenia during this observation window was recorded as the primary safety endpoint. "

**Reference:**

1 Jardim DL, Rodrigues CA, Novis YAS, Rocha VG, Hoff PM. Oxaliplatin-related thrombocytopenia. Ann Oncol. 2012;23(8):1937–1942. doi:10.1093/annonc/mds074.

3. Comment 3:

Non-inferiority is mentioned in the discussion - if the study was designed to test non-inferiority, please clearly indicate this in the sample size section of the paper.

**Response:**

Thank you for this important comment. We acknowledge that our study was not formally designed as a non-inferiority trial, and no non-inferiority margin was prespecified during the sample size calculation. To avoid confusion, we have removed the term “non-inferiority” from the Discussion section and have reframed our interpretation in terms of observed differences and clinical relevance. Please see lines 282–285 in the revised manuscript.

"However, as a pilot study with a limited number of patients, it lacks the statistical power to definitively demonstrate the superiority of biweekly CAPOX over triweekly CAPOX. Further studies are warranted to confirm the potential advantages of the biweekly regimen."

4. Comment 4:

What happened if a patient in the 3-month group experienced thrombocytopenia at 4 months - was that counted as an event? Be clear if the adverse events were collected within 6 months of starting treatment or just during study treatment.

**Response:**

Thank you for raising this point. Adverse events were systematically collected from the initiation of treatment until 28 days after the last dose of CAPOX, in accordance with standard clinical trial practice. Therefore, any thrombocytopenia occurring within 28 days after the final dose—including events in the 3-month group that happened during the 4th month—was included in the safety analysis. This time frame has been clarified in the Methods section of the revised manuscript (see lines 130–135 in the revised manuscript).

"Adverse events were monitored from the first day of treatment initiation until 28 days after the last dose of CAPOX. All events were assessed according to the Common Terminology Criteria for Adverse Events Version (CTCAE) version 4.0, and the incidence of all-grade thrombocytopenia during this observation window was recorded as the primary safety endpoint. "

5. Comment 5:

Figure 2F – state p > 0.99

**Response:**

We have updated Figure 2F and its legend to state 'p > 0.99'.

**R1 Adverse events in biweekly and triweekly groups.** (A) All grade thrombocytopenia in 3-month, 6-month and all patients between biweekly and triweekly group. (B) Grade more than 3 thrombocytopenia rate between biweekly and triweekly group. (C-D) All grade neutropenia and grade more than 3 neutropenia rate in 3-month, 6-month and all patients between biweekly and triweekly group. (E) Anemia rate in 3-month, 6-month and all patients between biweekly and triweekly group. (F) Peripheral sensory neuropathy rate in 3-month, 6-month and all patients between biweekly and triweekly group.

6. Comment 6:

The description of the results is valid.

Could 95% confidence intervals be added to the rates in Figures 2 and 3?

Response:

Thank you for your valuable comment. We have added the 95% confidence intervals (CIs) to the rates in Figure 2. Additionally, I have also provided the CI data for Figure 3 as shown in Figure R2 below for the detailed inclusion of the confidence intervals.

Furthermore, the description of the DFS data for both biweekly and triweekly groups, along with their respective 95% CIs, has also been included in the Results section, specifically on page 11, lines 197-212 in the revised manuscript.

" At the time of analysis, 29 events had been reported (13 in the biweekly group and 16 in the triweekly group). The Kaplan-Meier plots for DFS among biweekly group and triweekly group and subgroups are shown in Fig 3. The 3-year DFS rate was 85.1% [95% CI: 76.9%–93.3%] in biweekly group and 80.4% [95% CI: 71.2%–89.7%] in triweekly group (P = 0.51, HR = 0.78, [95%CI, 0.38-1.63]).

In the subgroup analysis of patients of 3-month treatment duration, the 3-year DFS was 89.2% [95% CI: 80.2%–98.2%] in biweekly group and 89.0% [95% CI: 79.8%–98.1%] in triweekly group. The difference was not statistically significant (P = 0.643). The HR for biweekly group to triweekly group was 0.89 [95% CI: 0.29–2.77], indicating no significant difference in DFS between the two groups.

For patients of 6-month treatment duration, the 3-year DFS was 77.6% [95% CI: 61.6%–93.6%] in biweekly group and 67.7% [95% CI: 50.3%–85.1%] in triweekly group, the HR was 0.79 [95% CI: 0.30–2.10] with no significant difference observed between the two groups ( P = 0.800). "

**R2 Adverse events and disease-free survival (DFS) in biweekly and triweekly groups.** (A) All grade thrombocytopenia in 3-month, 6-month and all patients between biweekly and triweekly group. (B) Grade more than 3 thrombocytopenia rate between biweekly and triweekly group. (C-D) All grade neutropenia and grade more than 3 neutropenia rate in 3-month, 6-month and all patients between biweekly and triweekly group. (E) Anemia rate in 3-month, 6-month and all patients between biweekly and triweekly group. (F) Peripheral sensory neuropathy rate in 3-month, 6-month and all patients between biweekly and triweekly group. (G) Overall disease-free survival (DFS) according to biweekly and triweekly CAPOX treatment groups.

## Reviewer #2

The authors present their single center randomized phase II study for high risk stage II and stage III colon cancer patients who received either biweekly or triweekly capeox. The rationale for the study is clear. The arms of the study are fairly well balanced and the results are presented appropriately. Limitations of the study are presented.

7. Comment 1:

Although T and N characteristics are presented it would be helpful to describe the numbers of Stage III A, B, C patients and high risk stage II patients and to describe which factors resulted in designating a stage II patient high risk.

**Response:**

Thank you for your valuable comment. In response to your request, I have provided a more detailed description of the Stage III A, B, C patients and high-risk Stage II patients in the manuscript. The proportions of Stage III A, B, C patients, as well as the breakdown of high-risk Stage II patients, are now detailed in Table 1.

For the definition of high-risk Stage II, we have followed the recommendations outlined in the NCCN guidelines. Specifically, the high-risk factors for Stage II are as follows:

1. Number of lymph nodes analyzed: If fewer than 12 lymph nodes were analyzed post-surgery.
2. Poor prognostic features: Poorly differentiated histology (exclusive of those that are MSI-H); Lymphatic/vascular invasion; Bowel obstruction; Perineural invasion (PNI); Localized perforation; Close, indeterminate, or positive surgical margins.

These factors have been clearly incorporated into the Results section, specifically on page 6, lines 96-101 in the revised manuscript.

Table 1 Characteristics of all enrolled patients

|  | Biweekly CAPOX  (n=80) | Triweekly CAPOX  (n=80) | P value |
| --- | --- | --- | --- |
| Duration of treatment |  |  | 0.74 |
| 6-month | 31 (38) | 29 (36) |  |
| 3-month | 49 (62) | 51 (64) |  |
| Age, median(range), years | 58 (32-77) | 57 (24-78) | 0.48 |
| <70 | 68 (85) | 71 (88) |  |
| ≥70 | 12 (15) | 9 (12) |  |
| Sex |  |  | 0.19 |
| Male | 45 (56) | 53 (66) |  |
| Female | 35 (46) | 27 (34) |  |
| Location of primary tumor |  |  | 0.19 |
| Right | 25 (31) | 22 (27) |  |
| Left | 52 (65) | 51 (64) |  |
| NA | 3 (4) | 7 (9) |  |
| T stage |  |  | 0.16 |
| T1-3 | 68 (85) | 61 (76) |  |
| T4 | 12 (15) | 19 (24) |  |
| N statge |  |  | 0.55 |
| N0 | 16 (20) | 17 (21) |  |
| N1 | 40 (50) | 45 (56) |  |
| N2 | 24 (30) | 18 (23) |  |
| Number of haversted lymph nodes |  |  | 0.32 |
| <12 | 2 (2) | 0 (0) |  |
| ≥12 | 70 (88) | 72 (90) |  |
| NA | 8 (10) | 8 (10) |  |
| TNM stage  Stage II  Stage IIIA  Stage III B  Stage III C | 20 (25)  36 (45)  15 (19)  9 (11) | 19 (24)  35 (44)  10 (12)  16 (20) | > 0.99 |
| CEA before surgery |  |  | 0.79 |
| <5 | 45 (56) | 47 (59) |  |
| ≥5 | 26 (32) | 22 (57) |  |
| NA | 9 (12) | 11 (88) |  |
| CA199 before surgery |  |  | 0.83 |
| <37 | 56 (70) | 54 (68) |  |
| ≥37 | 15 (19) | 15 (19) |  |
| NA | 9 (11) | 11 (13) |  |
| Recurrence patient | 13 (16) | 9 (11) | 0.35 |
| Uncomplete therapy patient | 6 (7) | 12 (15) | 0.13 |

8. Comment 2:

Although there were some differences in toxicities between the 2 regimens the differences are not striking and it is doubtful these results are practice changing.

**Response:**

Thank you for your valuable comment. We agree that the differences in toxicity between the two regimens are modest and not practice-changing at this stage. We acknowledge that the relatively small sample size in our study limits the ability to draw definitive conclusions regarding toxicity profiles. Nonetheless, our findings suggest that the biweekly regimen may offer a potential alternative for patients who have difficulty tolerating the triweekly oxaliplatin-based therapy.

While these results should be interpreted with caution, they do provide preliminary insights that may be clinically relevant for patient subsets with tolerability concerns. To address this in the manuscript, we have moderated our interpretation in the Discussion section. Specifically, we added the following sentences on lines 240-243 in the revised manuscript:

" Given the limited sample size, the observed differences in toxicity should be considered exploratory. Although not practice-changing, these findings may provide a rationale for future studies evaluating biweekly oxaliplatin regimens in patients with poor treatment tolerance. "

## Reviewer #3

The study titled "Biweekly CAPOX versus Triweekly CAPOX in the adjuvant therapy of post-surgery CRC: a randomized controlled trial" is a well-designed and clinically relevant investigation that addresses an important question in the management of colorectal cancer (CRC). The authors have conducted a randomized controlled trial to compare the safety and efficacy of a modified biweekly CAPOX regimen with the conventional triweekly CAPOX regimen in high-risk stage II and stage III post-surgery CRC patients. The study is particularly commendable for its focus on reducing treatment-related adverse events, such as thrombocytopenia and neutropenia, while maintaining therapeutic efficacy. The findings suggest that the biweekly CAPOX regimen is associated with fewer hematological adverse events and comparable 3-year disease-free survival (DFS) rates, which could have significant implications for improving patient tolerance and adherence to adjuvant chemotherapy. The study is well-written, methodologically sound, and provides valuable insights into optimizing adjuvant chemotherapy for CRC patients.

9. Comment 1:

While the study mentions that the sample size was calculated based on the primary outcome of thrombocytopenia, it would be beneficial to provide more details on the assumptions used for this calculation (e.g., expected incidence rates, effect size, power, and significance level). This will help readers understand the robustness of the study design and whether the sample size was adequate to detect clinically meaningful differences.

**Response:**

Thank you for your insightful comment. We appreciate the opportunity to clarify the assumptions used in the sample size calculation. The sample size was determined based on the primary outcome of all-grade thrombocytopenia.

The sample size calculation was based on the primary outcome of all-grade thrombocytopenia. Drawing upon both previous literature^1^ and our center's clinical experience, we assumed an expected incidence of 60% for the triweekly CAPOX group. For the biweekly CAPOX group, based on preliminary pilot data and clinical observations, we estimated a reduction to 39%. The effect size (Cohen’s h = 0.44) was calculated using the arcsine transformation formula for two proportions. With a two-sided significance level of 0.05 and a power of 80%, we determined that 80 participants per group were required.

We believe that this calculation supports the robustness of the study design, and while the sample size may limit the ability to detect very small differences, it is sufficient to identify clinically meaningful differences in thrombocytopenia incidence between the two regimens.

**Reference:**

1 Jardim DL, Rodrigues CA, Novis YAS, Rocha VG, Hoff PM. Oxaliplatin-related thrombocytopenia. Ann Oncol. 2012;23(8):1937–1942. doi:10.1093/annonc/mds074.

10. Comment 2:

The study reports a median follow-up period of 42 months, which is commendable. However, it would be useful to discuss whether this follow-up duration is sufficient to capture long-term outcomes, particularly for DFS and overall survival (OS). Consider providing a rationale for the chosen follow-up period and whether longer follow-up is planned.

**Response:**

Thank you for your thoughtful comment. We agree that the follow-up period is an important aspect of the study, especially when evaluating long-term outcomes such as disease-free survival (DFS) and overall survival (OS).

The reported median follow-up period of 42 months was chosen based on the expected time to observe meaningful differences in the primary endpoints^2^. Given that the majority of patients in our study had early-stage disease, we anticipated that this follow-up duration would allow us to capture key differences in DFS while still providing a reasonable assessment of OS in this patient population.

However, we acknowledge that longer follow-up may provide additional insights into the long-term effectiveness and potential late toxicities of the regimens. Our current study design does not extend beyond 42 months, but we plan to continue following these patients as part of an ongoing study to capture further long-term data. These extended follow-up results will provide a more comprehensive understanding of the clinical outcomes over a longer horizon. We hope this explanation clarifies the rationale behind the chosen follow-up period. If there are any additional questions or concerns regarding this, we are happy to address them.

**Reference:**

2 Yang L, Yang J, Kleppe A, Danielsen HE, Kerr DJ. Personalizing adjuvant therapy for patients with colorectal cancer. Nat Rev Clin Oncol. 2024 Jan;21(1):67-79. doi: 10.1038/s41571-023-00834-2.

11. Comment 3:

The study briefly mentions subgroup analyses based on treatment duration (3-month vs. 6-month). It would be valuable to provide more detailed results of these subgroup analyses, particularly in terms of adverse events and DFS. This could help identify specific patient populations that may benefit more from the biweekly regimen.

**Response:**

Thank you for your constructive feedback. We agree that providing more detailed results from the subgroup analyses based on treatment duration (3-month vs. 6-month) would be valuable, particularly in terms of adverse events and disease-free survival (DFS).

To address this, we have included the incidence rates of adverse events for both subgroups (3-month vs. 6-month) in the Adverse Events Table. This table provides a clearer comparison of the adverse events between the two treatment durations, which helps identify whether treatment duration influences toxicity profiles.

Additionally, we have updated the survival analysis by presenting separate Kaplan-Meier survival curves for the 3-month and 6-month subgroups. These curves, shown in Figure R3, illustrate the DFS for each subgroup, providing a clearer understanding of how treatment duration may impact long-term outcomes. We hope these updates will offer further insight into which patient populations may benefit more from the biweekly regimen. We have also further discussed these findings in the article, specifically in Result section on lines 203-212.

Table 2 Adverse events by regimen and treatment duration in all patients

| Adverse events | Biweekly CAPOX group  (n=80) | | | Triweekly CAPOX group  (n=80) | | | P value |
| --- | --- | --- | --- | --- | --- | --- | --- |
|  | 3-month treatment  (N=49) | 6-month treatment  (N=31) | total | 3-month treatment  (N=51) | 6-month treatment  (N=29) | total |  |
| Hematological |  |  |  |  |  |  |  |
| Neutropenia | 19(38) | 10(32) | 29(36) | 24(47) | 17(58) | 41(51) | 0.040 |
| Neutropenia (≥3) | 1(2) | 0 | 1(1) | 2(4) | 2(7) | 4(5) | 0.173 |
| Thrombocytopenia | 14(28) | 12(38) | 26(33) | 22(43) | 17(58) | 39(49) | 0.027 |
| Thrombocytopenia (≥3) | 1(2) | 3(10) | 4(5) | 5(10) | 2(7) | 7(9) | 0.349 |
| Anemia | 10(20) | 8(25) | 18(22) | 7(14) | 13(45) | 20(25) | 0.710 |
| Non-hematological |  |  |  |  |  |  |  |
| PNS | 24(49) | 16(52) | 40(50) | 32(62) | 15(52) | 47(58) | 0.267 |
| PNS (≥2) | 5(10) | 6(19) | 11(14) | 7(14) | 4(14) | 11(14) | 1.0 |
| Liver disfunction | 15(30) | 7(22) | 22(27) | 22(43) | 10(34) | 32(40) | 0.095 |
| Hyperbilirubinemia | 5(10) | 6(19) | 11(14) | 5(10) | 7(24) | 12(15) | 0.822 |
| Diarrhea (≥3) | 0 | 1(3) | 1(1) | 0 | 1(3) | 1(1) | 1.0 |
| Vomiting (≥3) | 0 | 0 | 0 | 2(4) | 0 | 2(2) | 0.155 |

**R3 Overall disease-free survival (DFS) and 3-year survival rate in subgroups.** (A) DFS according to biweekly and triweekly CAPOX treatment in 3-month group. (B) DFS according to biweekly and triweekly CAPOX treatment in 6-month group.

" In the subgroup analysis of patients of 3-month treatment duration, the 3-year DFS was 89.2% [95% CI: 80.2%–98.2%] in biweekly group and 89.0% [95% CI: 79.8%–98.1%] in triweekly group (Fig 4A). The difference was not statistically significant (P = 0.643). The HR for biweekly group to triweekly group was 0.89 [95% CI: 0.29–2.77], indicating no significant difference in DFS between the two groups.

For patients of 6-month treatment duration, the 3-year DFS was 77.6% [95% CI: 61.6%–93.6%] in biweekly group and 67.7% [95% CI: 50.3%–85.1%] in triweekly group (Fig 4B), the HR was 0.79 [95% CI: 0.30–2.10] with no significant difference observed between the two groups ( P = 0.800). "

12. Comment 4:

The study uses Kaplan-Meier estimation for DFS analysis and Cox proportional hazard models for hazard ratios (HRs). It would be helpful to clarify whether any adjustments were made for multiple comparisons, especially given the secondary endpoints. Additionally, consider discussing the potential impact of confounding variables on the results and whether any multivariate analyses were performed.

**Response:**

Thank you for your insightful comment. We appreciate the opportunity to clarify the points regarding adjustments for multiple comparisons and the potential impact of confounding variables.

Adjustments for Multiple Comparisons: In our study, we conducted comparisons across primary and secondary endpoints. However, we did not apply a specific adjustment for multiple comparisons (such as the Bonferroni correction) due to the exploratory nature of the secondary endpoints. While we acknowledge that adjusting for multiple comparisons could reduce the risk of Type I error, we feel that the results from the secondary endpoints still provide valuable insights that warrant further exploration in larger, confirmatory studies. We plan to consider appropriate adjustments in future studies with a more focused hypothesis.

Impact of Confounding Variables: We recognize that confounding variables, such as age, gender, comorbidities, and other treatment factors, may influence both the primary and secondary outcomes. To address this, we performed multivariate analyses, including adjustments for potential confounders, such as baseline patient characteristics and treatment variables, using Cox proportional hazards models. This approach allows us to better understand the independent effect of the treatment regimens on DFS and HR, controlling for these confounders. We hope this explanation addresses your concerns.

13. Comment 5:

While the study reports a lower incidence of thrombocytopenia and neutropenia in the biweekly group, it would be beneficial to provide more detailed information on the severity and management of these adverse events. For example, how many patients required dose reductions, treatment delays, or supportive care interventions?

**Response:**

Thank you for your valuable comment. We appreciate your suggestion to provide more detailed information on the severity and management of adverse events such as thrombocytopenia and neutropenia.

In response to your comment, we have now included additional details regarding the severity and management of these adverse events in the revised manuscript on lines 189-195. Specifically, we have provided data on the number of patients who required dose reductions and treatment delays due to thrombocytopenia and neutropenia. These findings have been incorporated into the Results section, where we provide a comprehensive summary of the adverse events and the corresponding management strategies in both treatment groups. We believe this addition enhances the clarity of the manuscript and provides a more complete picture of the treatment-related toxicities.

" Additionally, the proportion of patients requiring dose reductions was 13% in the biweekly regimen group and 28% in the triweekly regimen group. The proportion of patients with treatment delays was 10% in the biweekly regimen group and 26% in the triweekly regimen group, with the average treatment delay being 3 days and 5 days, respectively. These data highlight the differences in patient tolerance to the two treatment regimens and the challenges in managing adverse events. "

14. Comment 6:

The discussion section could be expanded to include a more detailed discussion of the clinical implications of the findings. For instance, how might the reduced incidence of hematological adverse events impact patient quality of life, treatment adherence, and overall healthcare costs? Additionally, consider discussing how these findings might influence clinical guidelines or practice.

Response:

Thank you for your valuable suggestion. We appreciate the opportunity to expand the Discussion section to include a more detailed analysis of the clinical implications of our findings.

We have now provided an expanded discussion on the impact of the reduced incidence of hematological adverse events, such as thrombocytopenia, in the biweekly regimen group. Specifically, we addressed how these reduced toxicities may improve patient quality of life, enhance treatment adherence, and reduce healthcare costs. Additionally, we have incorporated a more thorough analysis of how these findings might influence clinical guidelines and practice. These adjustments can be found in Discussion section on lines 254-270.

" In this study, we observed that the biweekly regimen was associated with a reduced incidence of hematological toxicities, including thrombocytopenia, compared to the triweekly regimen. While we did not directly assess hospitalization rates or healthcare costs, it is plausible that the reduction in toxicity may lead to improved patient quality of life by minimizing severe side effects, allowing for better physical function and fewer disruptions to daily activities. Additionally, the lower toxicity in the biweekly group might theoretically contribute to higher treatment adherence, as fewer patients required treatment discontinuation, dose reductions, or experienced treatment delays. Based on these observations, it is reasonable to speculate that the biweekly regimen could also offer a more cost-effective option by potentially reducing healthcare resources needed for managing severe toxicities. These findings suggest that the biweekly regimen could be a preferable treatment option for certain patient populations, particularly those at higher risk for adverse events. Ultimately, these results provide important insights that may influence clinical guidelines, offering a well-tolerated and effective alternative for patients who need continuous therapy. "

15. Comment 7:

The study acknowledges that it is a pilot study with a limited number of patients. It would be helpful to elaborate on other potential limitations, such as the single-center design, potential selection bias, and the generalizability of the findings to other populations or settings.

**Response:**

Thank you for your insightful comment. We appreciate the opportunity to address the potential limitations of this study.

As acknowledged in the manuscript, this study is a pilot study with a relatively small sample size, which inherently limits the statistical power and the ability to detect small differences between the treatment regimens. Additionally, we recognize that the single-center design may limit the generalizability of the findings. A single-center study often involves a more homogeneous patient population, which may not fully represent the broader population of patients with similar conditions. This could introduce selection bias and impact the external validity of the results.

Furthermore, due to the nature of this study, we are cautious about generalizing the findings to other healthcare settings, particularly those with different patient demographics or treatment practices. The results may be more applicable to similar settings with comparable patient populations and treatment protocols. Future multicenter studies with larger sample sizes will be essential to confirm these findings and assess their applicability across diverse patient groups.

We have acknowledged these limitations in the Discussion section (see lines 282-293) and emphasize the need for further research to validate these preliminary results in more diverse populations and settings.

"However, as a pilot study with a limited number of patients, it lacks the statistical power to definitively demonstrate the superiority of biweekly CAPOX over triweekly CAPOX. Further studies are warranted to confirm the potential advantages of the biweekly regimen. Additionally, this study is limited by its single-center design, which may introduce potential selection bias and limit the generalizability of the findings to other populations or healthcare settings. The sample size and setting may not fully represent the broader patient population, and the results may not be applicable to patients with different demographic characteristics or in different clinical contexts. As such, while our findings provide important insights, larger, multi-center studies are needed to validate these results and to assess the broader applicability of the biweekly regimen across diverse patient groups. "

16. Comment 8:

The conclusion could be strengthened by suggesting directions for future research. For example, are there plans for a larger, multicenter trial to confirm these findings? Could future studies explore the use of biomarkers or molecular profiling to further personalize adjuvant chemotherapy regimens?

**Response:**

Thank you for your valuable suggestion. We agree that the conclusion could be strengthened by outlining potential directions for future research.

To confirm the findings from this pilot study, we plan to conduct a larger, multicenter trial that will involve a more diverse and representative patient population. This will help address the limitations of the current study, such as its small sample size and single-center design and provide more robust data to validate the results.

Additionally, future studies could explore the use of biomarkers or molecular profiling to further personalize adjuvant chemotherapy regimens. By identifying specific molecular characteristics or biomarkers associated with treatment response or toxicity, we could better tailor treatment plans to individual patients, improving both efficacy and minimizing adverse effects. This would be particularly valuable for optimizing therapy in high-risk populations and ensuring that patients receive the most effective treatment with the least amount of toxicity.

We have included these potential directions for future research in the Conclusion section (see lines 295-300) to highlight the next steps in advancing this field.

"Our pilot study suggests that the biweekly CAPOX regimen may be a viable alternative to the triweekly regimen, especially for patients with oxaliplatin intolerance. However, these findings need to be confirmed in larger, multicenter trials. Future research could also explore the use of biomarkers or molecular profiling to personalize adjuvant chemotherapy regimens and optimize treatment outcomes. "
